# K_V_11.1, Na_V_1.5, and Ca_V_1.2 Transporter Proteins as Antitarget for Drug Cardiotoxicity

**DOI:** 10.3390/ijms21218099

**Published:** 2020-10-30

**Authors:** Magdalena Kowalska, Jacek Nowaczyk, Alicja Nowaczyk

**Affiliations:** 1 Department of Organic Chemistry, Faculty of Pharmacy, Collegium Medicum in Bydgoszcz, Nicolaus Copernicus University, 85-094 Bydgoszcz, Poland; magda.kowalska@doktorant.umk.pl; 2 Faculty of Chemistry, Nicolaus Copernicus University, 87-100 Toruń, Poland; jacek.nowaczyk@umk.pl

**Keywords:** antitarget, drug cardiotoxicity, ion transporter proteins

## Abstract

Safety assessment of pharmaceuticals is a rapidly developing area of pharmacy and medicine. The new advanced guidelines for testing the toxicity of compounds require specialized tools that provide information on the tested drug in a quick and reliable way. Ion channels represent the third-largest target. As mentioned in the literature, ion channels are an indispensable part of the heart’s work. In this paper the most important information concerning the guidelines for cardiotoxicity testing and the way the tests are conducted has been collected. Attention has been focused on the role of selected ion channels in this process.

## 1. Introduction

Safety Pharmacology is one of the most dynamically developing disciplines, whose objective is to assess the potential risks of improperly conducted pharmacotherapy. Evaluation of the safety in the use of a substance is a key part of placing a new medicine on the market. The Organization for Economic Co-operation and Development (OECD) has proposed a number of guidelines for drug safety testing [1,2]. However, the continuous development of medicine allows the extension and refinement of the test panel that a new molecule must undergo before it can be released for use by patients. Toxic effects of compounds on the most important organs is one of the frequent reasons for eliminating substances from further tests. Additionally, safety tests are also performed for medicines already approved for use. Such trials are required, e.g., to register new indications for “old” drugs.

All abovementioned activities require a suitable definition of the antitargets. They are defined as undesirable molecular targets that play an essential role in the proper functioning of cells. Down modulation of an antitarget results in clinically unacceptable side effects, initiation of disease, or deleterious alterations in disease progression. This results in shorter onset time of the disease, increased disease burden, poorer patient outcome, or decreased survival time.

The amphiphilic nature of lipid molecules, which contain both polar and hydrophobic parts, determines the weak permeability of lipid bilayers for broad range of substances and thus allows the membranes to perform barrier functions effectively in the cells. Since the lipid bilayer of cell membranes is almost impermeable to ions, their transport is possible through specialized transmembrane transport proteins called ion channels. These are defined as macromolecular pores made of many protein subunits through which ions can passively move through the cell membrane [3]. More than 650 types of human ion channels have been identified so far [4,5,6]. Additionally, scientists have proven that different types of ion channels make up about 1.5% of the human genome [7]. Ion channels are the third-largest target for existing drugs (after G-protein coupled and nuclear receptors). When the channel is open, there is little interaction between the channel protein and the types of ions that pass through it, since the channel itself does not undergo conformational change to allow the ions to pass through [3]. It is the fastest type of transport protein, allowing movement at up to 10^8^ ions per second, which is equal to electric current of a few picoamperes (10^−12^A) generated by the ionic flow through a single open channel [8]. Commonly the magnitude of potential difference across the cell membrane of living cells is 10^−3^ V. This enables cells to communicate quickly and play a fundamental role, allowing vital functions, such as the brain receiving and processing information, the heart beating, and muscles working [7]. Therefore, ion channels play a crucial role in several important physiological and pathological processes.

Since many heart diseases result from disruption of the normal ion channel function, this work is devoted to reviewing selected antitargets for cardiotoxicity. This article reviews the transport proteins K_V_11.1, Na_V_1.5, and Ca_V_1.2 as potential undesirable drug targets. Recent topics related to in silico cardiotoxicity studies are presented in a concise form. The review includes a historical overview of the cardiotoxicity of drugs, the functions, and the structure of K_V_11.1, Na_V_1.5, and Ca_V_1.2. Considering the wide application of the molecular docking technique, the key properties of the structural subunits of the studied heart ion channels and a set of selected drugs inhibitory potencies values are gathered.

## 2. Historical Overview of Drug Cardiotoxicity

**Figure 1:**
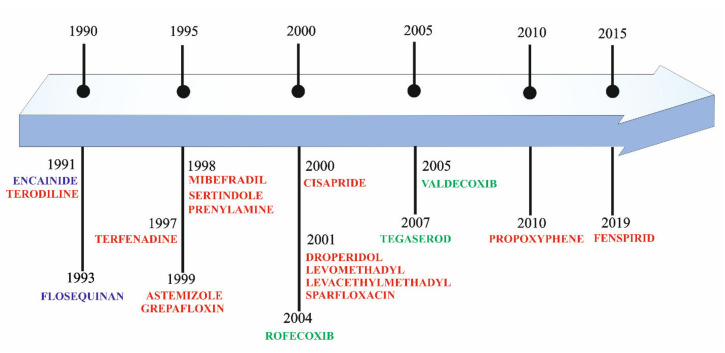
Drugs withdrawn from market due to proarrhythmic effect (purple), myocardial infarction (green), torsadogenic potential, i.e., QT interval prolongation and torsade the points (TdP) effect (red).

The statistics show that the risk of cardiotoxicity is one of the most common reasons for withholding or withdrawing drugs from the market [9,10]. This unwanted effect is shared by a large number of non-cardiovascular drugs. The statistics show that up to 70% of potential medicines are either not approved for further testing or their use is limited [11]. Additionally, it has been estimated that approximately 2–3% of all drug prescriptions involve medications that may unintentionally cause long QT syndrome [12]. The first drugs that were removed from clinical use due to cardiotoxicity were encainide (proarrhthmic effect) and terodiline (QT interval prolongation) (Figure 1) [13,14]. Terdynafine, cisaprid, astemizole, sertindol, thoridazine, grepafloxacin have been removed due to heart toxicity [15] (Figure 1). In 2020, there was information about QT prolongation for other drugs: osilodrostat, carbetocid, selpercatinib, and rucaparib [16]. In 2005, the Council on Harmonization of Technical Requirements for Registration of Pharmaceuticals for Human Use suggested principles for checking new molecules for the risk of inducing potentially fatal arrhythmias (e.g., torsade the points (TdP)) caused by blocking the human Ether-à-go-go-Related Gene (hERG, K_V_11.1) [17,18]. The first antitarget in compound cardiotoxicity studies was thus defined [11]. This was based on the observed correlation between electrocardiogram QT prolongation and patients’ risk of TdP arrhythmia. The preclinical and clinical studies proposed in S7B and E14 were implemented and are now widely used in the pharmaceutical industry and regulatory agencies [17,18]. These assumptions were accepted as the crucial elements of the compound cardiotoxicity assessment. However, subsequent years of research have demonstrated that proarrhythmic effect, QT prolongation, and hERG blocking cannot be treated as the only determinants of the occurrence of TdP [19]. Verapamil and ralonazine are examples of drugs that are strong inhibitors of the hERG channel and at the same time devoid of the risk of inducing arrhythmias and vice versa, serious disorders of cardiomyocyte electrophysiology caused by drugs that are weak hERG inhibitors (e.g., sotalol, alluzosin) [20,21]. Thus, it proves the insufficient specificity of the tests based only on the assessment of the hERG channel blocking potential [22]. The risk of drug-induced TdP is rather balanced by multiple internal cardiac ionic currents that define ventricular repolarization [23]. Thus, for the first time in 2013, a new strategy was applied for a real proarrhythmic risk assessment of molecules: Comprehensive In Vitro Proarrhythmia Assay (CiPA). CiPA is utilized in the first stages of drug research and focuses on the three most important areas. The first one is the assessment of the interaction of molecules with three ion channel models. In addition to studying the interaction of molecules with hERG, CiPA proposes two additional ion channels gated by voltage, which are essential for the development of arrhythmia: Na_V_1.5., Ca_V_1.2. The second area of CiPA concerns in silico simulations of action potential (AP) responsible for arrhythmia. The third area of CiPA’s research is advanced cellular testing using cardiomyocytes derived from pluripotent stem cells (iPSC-CM) [24].

## 3. VGICs—Voltage-Gated Ion Channels

The mechanism of opening and closing of ion channels is referred to as gating. It is the progression of the channel through various conformational states. Channel activation is the transition from the rest (closed) state to the open state under the influence of a trigger stimulus (membrane depolarization or ligand binding). Permeation is the passage of ions through the open channel. Therefore, gating process control ion permeation [8] participating in cellular signaling is dependent on cation flow driven by electrochemical gradient. Voltage-gated Na^+^, Ca^2+^, and K^+^ channels share a common molecular architecture. They also possess the same set of three voltage-dependent functionally distinct states, i.e., closed (or resting), activated (or open), and inactive [12]. They are divided into two types: selective towards one type of ion (such as potassium, sodium, or calcium channel) and non-selective, which are capable of transporting any type of ion through the membrane (e.g., N-methyl-D-aspartate receptor) [25]. The important determinants of selectivity are size, valency, and hydration energy [5]. Under physiological conditions, the Na^+^ and Ca^2+^ channels are inward cell currents and those flowing through the K^+^ channels are outward currents. Besides the channels that open or close depending on the value of the cell transmembrane potential (so-called voltage-gated ion channels (VGICs)), there are also channels gating independently on transmembrane potential, controlled by other external or intracellular factors.

According to the research of International Union of Primary and Clinical Pharmacology and the British Pharmacological Society, ion channels are the third-largest target group for drugs [26,27]. They participate in controlling physiological and pathological processes in the body. VGICs take part in generating and transmitting information within the cells of the central and peripheral nervous and cardiovascular systems [25]. For example, it has been estimated that about 350 types of ion channels exist in the mammalian brain, including 145 VGICs [28]. At the root of certain diseases (e.g., neuropathic pain or epilepsy) is abnormal functioning of the channels [29,30]. In the recently proposed changes to the cardiovascular safety assessment paradigm (CiPA initiative), inter alia, by indicating the need to assess drug interactions with cardiac VGIC (such as: K_V_11.1, Na_V_1.5, and Ca_V_1.2) and their impact on the electrophysiology of human ventricular cells using in silico and/or in vitro tests. It should be emphasized that these three classes of ion channels not only represent useful targets with potential therapeutic applications, but also indicate undesirable targets (also known as antitargets) to be avoided because of the side effects they cause when their function is altered [31].

## 4. VGICs—Structure

**Figure 2:**
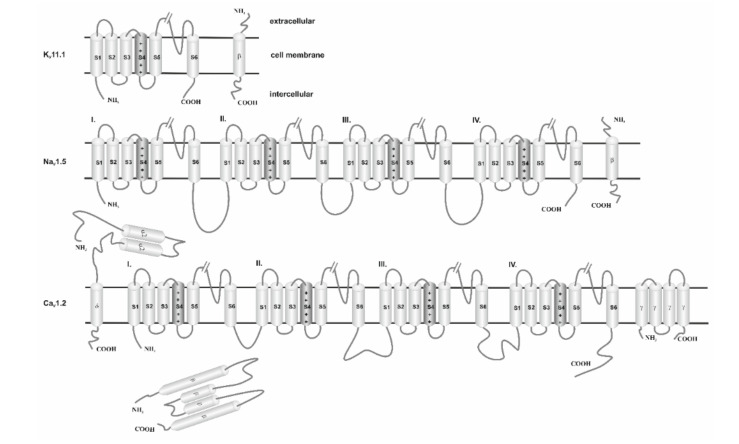
Schematic view of the structure’s subunits of cardiac ion channels. α1−subunits of K^+^ channels consist of one single subunit (domain or core motif), while Na^+^ channels and Ca^2+^ channels consist of four serially-linked homologous domains (I–IV). Each subunit contains six transmembrane segments (S1–S6). The S5 and S6 segments and the membrane-associated pore loop (often called the P loop or P segment or P region) between them form the central pore through which ions flow down their electrochemical gradient. The S4 transmembrane domain is the voltage-sensor (gating modifier). K^+^ channel is a tetramer assembly of α subunits. While Na^+^ and Ca^2+^ channels consist of four subunits co-assembled to form a single functional channel. The β, α2δ, and γ subunits enhance cell surface expression and modulate the voltage dependence and gating kinetics of the α1 subunits and channel sensitivity to endogenous ligands and pharmacological agents. These accessory subunits determine ion channel tissue specificity.

The molecular architecture of the voltage-gated ion channel families consists of four subunits arranged to form a common pore structural theme. Ion channels consist of pore-forming α-subunits (also called α1-subunits) and accessory α2−, β−, δ−, γ-subunits. The α-subunit is inserted into the lipid bilayer of the cellular membrane and constitutes the channel pore through which ions pass (Figure 2). Commonly, α-subunits and accessory subunits are members of large protein families that evolutionarily possess similar structural elements, i.e., comparable amino acid sequences. This is reflected in the names of the subunits and their genes. For example, the gene encoding the α-subunit of the cardiac Ca^2+^ channel is called CACNC1C: calcium channel, isoform 1, α-subunit. The α-subunit is termed Ca_V_1.2: Ca^2+^ channel family, subfamily 1, member 2; the subscript “V” means that channel gating is regulated by transmembrane voltage changes (voltage dependent) [32].

### 4.1. Potassium Ion Channels

The largest and most diverse group of cationic ion channels is those transporting potassium ions. It has been established that K^+^ channels occur in the plasma membrane of almost all animal cells. Due to this, they represent a large family of therapeutic targets/antitargets for drug development. According to the literature, the human genome encodes 40 voltage-gated potassium channels [7], which are involved in diverse physiological processes ranging from repolarization of neuronal or cardiac APs, over-regulating calcium signaling and cell volume, to driving cellular proliferation and migration. This group includes 12 subfamilies, located in the brain, heart, and muscles (K_V_1–K_V_12) [28,33]. The main building blocks of these proteins are α subunits, which contain pores selective in potassium ions. Both the amino terminus (N-terminus) and the C-terminus of α subunits are located on the intracellular side of the membrane (Figure 2 and Table 1). Interaction of K_V_11.1 with its β-subunit, i.e., Mink-related peptide 1 (MiRP1, encoded by KCNE2) [34] induces earlier activation and accelerates deactivation. In this way the ancillary protein (UniProtKB references code: Q9Y6J6) [35] modulates the gating kinetics and enhances stability of the channel complex. It has a single transmembrane segment, a long extracellular N-terminus, and a short intracellular C-terminus [36] (Figure 2).

The K_V_11.1 channel (encoded by the hERG gene) is the best-known potassium ion channel. Brief characteristic membrane topology and structural organization of the K_V_11.1 subunit are presented in Figure 2 and Table 1. In the last decade of the twentieth century it was found that K_V_11.1 plays a crucial role in cardiac repolarization, especially in the later phases of the AP based on its unique kinetics. KCNH2 gene codes for the K_V_11.1 channel, known as the human Ether-à-go-go-Related Gene (hERG), carries the delayed rectifier potassium current (IKr). IKr is a key component in repolarization of the myocardium [38]. Opening of the channel and rapid transport of potassium ions driven by the electrochemical potential gradient occur just after depolarization of the cell membrane, in the first stages of functional potential. Repolarization results in a reopening of the channel, resulting in the termination of the potential/excitation [39]. For this reason, the hERG current is the most common target for QT interval-prolonging drugs [40]. Drugs blocking the hERG potassium channels (referred to as “hERG inhibitors’’) reduce the IKr and prolong cardiac repolarization, which appears as prolongation of the heart rate-corrected QT (QTc) interval on the electrocardiogram (ECG), and this predisposes arrhythmias [41]. Therefore, it is commonly well accepted as an antitarget in cardiac risk assessment. Moreover, these channels have also been identified in neurons, thus explaining the fact that a disruption of Kv activity may also lead to epilepsy [30,39]. The function of the hERG gene-coded channel can be impaired by a number of drugs. One of them is terfenadine (TNF), a second-generation antihistamine [42]. According to numerous studies, the combination of TNF with the protein encoded by the hERG gene is a direct cause of prolonged QT and thus strong ventricular arrhythmia [43]. Consequently, the use of TNF was abandoned in the USA in favor of its active metabolite, fexofenadine [44]. However, the strong interaction between TNF and K_V_11.1 is a benchmark in testing the inhibitory power of the K_V_11.1 channel relative to other drugs.

### 4.2. Calcium Ion Channels

The role of calcium in normal heart activity was first mentioned in 1883 [45]. In the 1990s, there was evidence of the effect of Ca^2+^ ions on muscle cells [46]. Nowadays, it is widely acknowledged that calcium is involved in many cellular processes. The Ca^2+^ channel family contains at least ten members that are distinguished by their structure, subunit composition, location, biophysical properties, and pharmacology. By controlling the entry of Ca^2+^ into cells, these proteins have a critical role in a broad range of cellular processes, such as neurotransmitter release, second messenger cascades, cardiac excitation and contraction, and gene regulation supporting learning and memory [47]. The studies indicate that calcium ion channels are involved in myocardial contraction, hormonal regulation, and nervous system function. Voltage-gated Calcium Channels (VGCaCs) are complex proteins consisting of four or five subunits. The channel is built of one central pore-forming α1 subunit, five β subunits, four α2δ subunits, and five γ subunits [31]. The most important α1 subunit is responsible for the major biophysical and functional properties of the channel, while the auxiliary subunits α2δ, β, and γ control channel expression, membrane incorporation. Those auxiliary subunits are also involved in the drug binding and gating characteristics of the central unit (Figure 2). Drugs that block Ca^2+^ channels are used in the treatment of epilepsy, chronic pain, and cardiovascular disorders (including hypertension, angina pectoris, and cardiac arrhythmias) [48]. The α2 subunit is completely extracellular, whereas the δ subunit has a single membrane-spanning segment with a very short intracellular part that anchors the α2δ subunit (encoded by CACNA2D1-4) complex to the α1C subunit. Both α2 δ subunits are disulfide-linked proteins. The β subunit (Cavβ1- Cavβ4, encoded by the CACNB1-4 gene) is entirely intracellular and is tightly bound to a highly conserved motif in the cytoplasmic linker between domains I and II of the α1C subunit [5]. The γ subunit is composed of four transmembrane segments and intracellular N- and C-termini (Figure 2.).

The location of VGCaCs in many different cells has resulted in the classification of the currents according to the rate of ion conduction within the channel. Long opening currents are observed after strong depolarization, mainly within muscle and hormone cells. They are inhibited by drugs such as dihydropyridine or benzodiazepines. They take part in the initiation of the neurotransmission. T-type currents are initiated when a slight depolarization occurs. These currents are short-term and resistant to the abovementioned blocking substitutions, while they are blocked by mibefradil (withdrawn from treatment for side effects, Figure 1) [49]. According to current differences three groups of ionic channels have been distinguished: Cav1 mediating the transfer of L-type currents, Ca_V_2 mediating the transfer of N-, P/Q-, and R-type currents, Ca_V_3 mediating the transfer of T-type currents. In cardiac muscle, two types of voltage-dependent Ca^2+^ channels, the L-type and the T-type, transport Ca^2+^ into the cells.

VGCaCs can exist in a resting, open, or inactive form. The opening of the ion channel occurs during the depolarization of the cell membrane, which leads to an inflow of calcium ions into the cell and its excitation. Thus, depolarization leads to the transition from inactive to open state; however, it can be modified by the action of neurotransmitters or hormones [50,51].

The Ca_V_1.2 channels dominate the functional activity in the working myocardium [51,52]. Additionally, Ca_V_1.2 belong to the L-type Ca^2+^ channels and are critical to maintain the action potential plateau, to accelerate pacemaker activity in the sinoatrial node, and to support conduction through the atrioventricular node. Because of their importance in the normal cardiovascular function, screening of new drug candidates for their activity on Ca_V_1.2 channels is considered an important safety measure in developing new pharmaceuticals which are devoid of undesirable cardiovascular side effects [53]. Malfunction or mutation of the gene encoding Ca_V_1.2 can lead to the occurrence of diseases (e.g., Timothy syndrome), which are manifested by the QT- prolongation [54]. Moreover, a sustained increase in the inflow of ions to the cell due to channel hyperactivity leads to hypertrophy of the myocardium, which results in failure and hypertension. The Ca_V_1.2 channels play a dominant role in peripheral vasoconstriction and are the target of Ca2+ channel blockers used to treat hypertension [40]. The studies conducted in 1960 resulted in the discovery of the VGCaC blockade mechanism, allowing for the development of drugs inhibiting the flow of Ca^2+^ ions [55]. Three groups of drugs are commonly used in the treatment: nifedipine, verapamil, and diltiazem. The mechanism of action of these drugs is based on the blocking of L-channels that lead to vasodilation [47].

### 4.3. Sodium Channels

Voltage-gated sodium channels (VGNaCs) were first described in the electric eel *Electrophorus electricus* in 1976 [56]. They became the model on which the whole group of ion channels was characterized. They initiate APs in nerves, muscles, and other electrically excitable cells [57]. Blocking VGNaCs makes these cells less excitable [58]. Eukaryotic VGNaCs are composed of α and β subunits. Subunit α contains pore-forming and voltage-sensing domains to control the penetration of Na+ ions through the membrane. Subunits α are encoded by the SCNXA gene (where X = 1 − 9, depending on the ion channel type). Auxiliary subunits β modulate gating and regulate the channel expression [59]. So far, four subunits β (β1–β4) have been identified [57,59]. β-Subunits have a single transmembrane segment, a long extracellular N-terminus, and a short intracellular C-terminus. Presently, there are nine different types of α subunits, from which individual ion channels (Na_V_1.1–Na_V_1.9) have been isolated. So far, Na_V_1.5 is the best studied channel, which is the most common in myocardial cells.

VGNaCs are important targets for the development of drugs, because mutations in different human sodium channel isoforms have causal relationships with a range of neurological and cardiovascular diseases [60,61].

Depending on the location, the channels have different functions. Na_V_1.1–Na_V_1.3 are most abundant in the Central Nervous System. They are the therapeutic target of several drugs in pain, stroke, or migraine (Nav1.1). This location also contains Nav1.6 channels, which are used to treat multiple sclerosis. The proper activity of the musculoskeletal system is regulated by the Nav1.4 channel. Nav1.7–1.9 function mainly in the peripheral nervous system, used to treat pain and nociceptive disorders [62].

**Figure 3:**
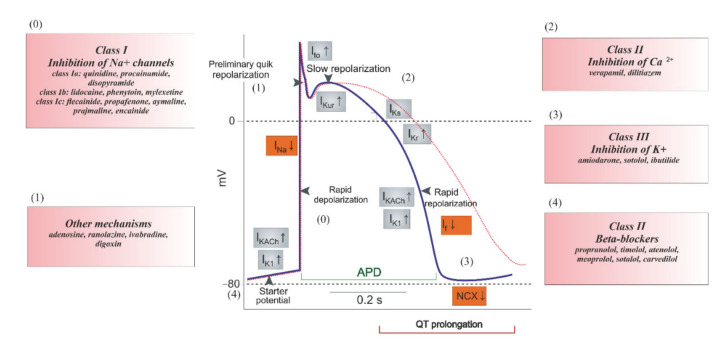
Classification of cardiac antiarrhythmic drugs.

Dysfunction of VGNaCs can lead to a number of problems. Until now, more than 1000 disturbances caused by mutations in the Na_V_ channels have been identified. It should be noted that about 400 diseases are caused by a mutation of the Na_V_1.5 gene [63]. Moreover, the channel Na_V_1.5 (next to Na_V_1.2) has the highest number of reported mutations among all nine Na_V_ channels. Mutations in Na_V_1.5 result in many cardiac channelopathies [64]. Mutations leading to a reduction of the sodium current can result in disorders such as Brugada syndrome, sick sinus syndrome, and cardiac conduction defect and others. Strengthening the function of the aforementioned channel is a leading cause of the occurrence of sudden infant death syndrome and stillbirth, whereas the reason for arrhythmias and prolonged QT can be both stimulating and inhibiting Na_V_1.5 activity [63,65]. Recent evidence suggests that a failure of the channels Na_V_1.1-Na_V_1.3 and Na_V_1.6 can lead to epilepsy or maintenance of the epileptic state [60]. Current scientific papers emphasize that Na_V_1.7 overactivity can determine the pain sensation even when sympathetic neuronal excitability is reduced [66]. In turn, Na_V_1.8 and Na_V_1.9 take part in setting up inflammatory pain [67]. Nonetheless, there are a multitude of substances used to control VGNaCs activity by blocking the sodium channels. According to the above, abnormal inflow and load of Na^+^ is associated with neuronal damage. Tetradotoxin and batrachotoxin, which are naturally occurring toxins, strongly block the activity of sodium channels [60,68]. Therefore, drugs have been elaborated to treat diseases caused by overactivation of VGNaCs. The most commonly used drugs are first-generation antiarrhythmic medications and those used to treat epilepsy (e.g., lamotrigine, phenytoin, or carbamazepine) [69]. The drugs used in arrhythmia are listed in Figure 3 [70]. On the other hand, it is important to avoid interactions of potential non-cardiovascular drugs on Na_V_1.5, as well as hERG due to potential off-target activity [63].

## 5. Mechanism of Ion Channel Inhibition

Although the general mechanism of ion channel inhibition is well known, the detailed description is still unclear and controversial. Voltage-dependent gating can be triggered in a variety of ways, and the mechanisms of VGIC operation are important tools to understand the signaling behavior of the channel [71]. The mechanisms of ion channel inhibition can be categorized in two classes, i.e., pore plugging, and allosteric binding. The former includes inhibitors capable of binding in the pore region once they enter the channel; in consequence they physically block the pore disabling the ion transport. The latter group is inhibitors that require a specific binding site, the site is usually an extracellular side of the pore, but there are known exceptions. The allosteric inhibitor binds to the channel at the binding site causing conformational changes of the protein that prevents the normal function of the channel [12]. Table 1 summarizes the pore forming region in K_V_11.1, Na_V_1.5, and Ca_V_1.2 channels.

## 6. In Silico Methods for Testing the Risk of Cardiotoxicity

One of the most popular and accurate in-silico methods is the molecular docking technique [72]. This method determines the affinity (the binding of compounds to the channel) and how a given drug binds to the active site of a protein (also known as binding modes/pose). It is a source of information about the physiological and pathological mechanism of action of many substances [73]. It should be mentioned here that channels K_V_11.1, Na_V_1.5, Ca_V_1.2 can be treated in two ways. First, they are therapeutic targets for cardiac arrhythmia and hypertension. Secondly, they should be treated as antitargets in cardiac risk assessment. This entails the necessity of opposite approaches in interpretation of the channel’s blocking effects. In the first case, research is conducted to search for molecules that bind to a specific therapeutic target (e.g., Class III antiarrhythmic drugs). In the second, research is focused on looking for molecules that avoid strong binding to the unwanted targets (i.e., many noncardiac drugs) [11,74]. Commonly, K_i_ is characterized by the dissociation constant of the complex, which is determined by competitive radioligand binding, and the blocking of the passage of ions through the channel. In the computational approach, K_i_ is determined in the computational process. In vivo desirable and undesirable effects of a drug are generally related to its concentration at the sites of action [75]. The passage of some ions through the channel is characterized by the IC_50_ concentration, ensuring a 50% reduction in selected ion current, which is determined by electrophysiological methods in the channel-expressing cells. For the majority of compounds, only data obtained by one of experimental methods are available. It should be emphasized here that the values of these parameters are generally well correlated (IC_50_ ≈ K_i_). Based on the assumption that the calculated K_i_ reflects the drug binding affinity for a specific therapeutic target and the IC_50_ (or pIC_50_) more reflects the functional potency of the inhibitor for the drug, it is widely accepted that the use of calculated K_i_ (or pK_i_) is helpful in determining the likelihood that a particular drug will inhibit a particular protein target and result in a clinically relevant drug interaction. For this we usually assume that the IC_50_ corresponds to the numerical value of K_i_ (or pIC_50_ ≈ pK_i_). By convention, a pIC_50_ ≤ 4 (or pK_i_) is commonly used as threshold and can be considered to be an inactive compound for the studied protein [76,77]. In Table 2, pIC_50_ of selected drugs and corresponding ion channels were collected. By matching the calculated K_i_ values with the corresponding data from Table 2, we are able to assess, for example, the cardiotoxicity of drugs by analyzing their interaction with ion channels [78]. From a safety pharmacological point of view, lower pIC_50_ (pK_i_) value for channels K_V_11.1, Na_V_1.5, Ca_V_1.2 are desirable.

## 7. Conclusions and Perspective

Despite the fact that the electrical excitability of nervous and muscle tissue has attracted the attention of scientists for three centuries, many issues remain unclear and controversial to this day. Of course, the substantial development in this area cannot be overlooked! It is worth emphasizing that since the mid-1990s, researchers have been addressing issues related to the cardiological safety of drugs that are being developed and already used. In the past 25 years, pro-arrhythmic risk testing has dominated the assessment of cardiovascular safety and the discipline of safety pharmacology. The regulatory guidelines (clinical and non-clinical) developed so far have focused mainly on inhibition of the human Ether-à-go-go-Related (hERG) gene and pulse-corrected QT prolongation (QTc). However, in our opinion, the science of cardiac safety must evolve, and this evolution should be multidirectional. Undoubtedly, modern technology creates a multitude of different possibilities, the use of which should create conditions for a better understanding of the pharmacology of cardiac safety. Unquestionably, in silico tests based on the molecular docking technique are one such possibility. The key seems to be proper understanding and differentiation of concepts such as desirable and undesirable therapeutic goals. On the other hand, however, we should be aware that, under certain therapeutic conditions, our therapeutic targets become primary antitargets (i.e., undesirable targets) in drug development. Antitargets are molecular systems that play an essential role in the normal functioning of cells and tissues and their blockage causes potentially serious side effects. This results in poorer treatment outcomes or a shorter patient survival time. Since many drugs can bind to ion channels, block ion flow, and interfere with the regulation of the action potential, leading to drug-induced arrhythmias or “proarrhythmias”, these structures appear to be appropriate antitargets in assessing cardiac risk.

## Figures and Tables

**Table 1:** Overview of the key properties of the structural subunits of cardiac ion channels [37].

Name	hK_V_11.1/KCNH2	hNa_V_1.5/SCN5A	hCa_V_1.2/CACNC1C
UniProtKB	Q12809	Q14524	Q13936
pore-forming	612–632: VTALYFTFSSLTSVGFGNVSP	884–904: FFHAFLIIFRILCGEWIETMW	694–715: QSLLTVFQILTGEDWNSVMYDG
ion selectivity sequence motif	GYG	DEKA	EEEE
cariac disease	Long Qt Syndrome; Short Qt Syndrome	Atrial Fibrillation, Familial, Brugada Syndrome; Cardiomyopathy, Dilated; Long Qt Syndrome; Progressive Familial Heart Block (Type Ia); Sick Sinus Syndrome, Autosomal Recessive; Sudden Infant Death Syndrome; Ventricular Fibrillation During Myocardial Infarction, Susceptibility to acquired arrhythmia	Long QT, Brugda Syndrome, Timothy Syndrome
sequence identity ^1^	88.66%	66.70%	70.3%

^1^ Sequence identity between the template and the modeled sequence.

**Table 2:** Comparison of pIC_50_ values (i.e., the negative logarithm of the IC_50_ value) of selected drugs.

Drug	hNa_V_1.5	hCa_V_1.2	hK_V_11.1
ajmaline	5.09 [79]	4.15 [80]	5.98 [81]
amiodarone	5.32 [82]	5.57 [83]	7.52 [21]
amitryptyline	4.70 [84]	4.94 [85]	5.48 [86]
bepridil	5.43 [21]	6.68 [21]	7.48 [87]
chlorpromazine	5.37 [88]	n/a [88]	5.83 [89]
cibenzoline	5.11 [90]	4.52 [91]	4.65 [92]
cisapride	4.83 [21]	n/a [21]	8.19 [93]
desipramine	5.82 [21]	5.77 [21]	5.86 [94]
diltiazem	5.05 [95]	6.35 [96]	4.76 [97]
diphenhydramine	4.39 [21]	3.64 [21]	5.28 [92]
dofetilide	3.52 [98]	4.22 [99]	8.30 [100]
fluvoxamine	4.40 [21]	5.31 [21]	5.51 [101]
haloperidol	5.15 [102]	5.77 [102]	7.57 [103]
imipramine	5.44 [88]	5.08 [89]	5.47 [101]
mexiletine	4.37 [104]	4.00 [105]	4.30 [106]
mibefradil	6.01 [107]	6.81 [108]	5.74 [92]
nifedipine	4.43 [88]	7.22 [109]	3.56 [110]
nitredypine	4.44 [88]	9.46 [111]	5.00 [101]
phenytoin	4.31 [21]	3.99 [21]	4.00 [101]
pimozide	7.27 [112]	6.79 [113]	7.70 [101]
prenylamine	5.60 [21]	5.91 [21]	7.19 [21]
propafenone	5.92 [21]	5.74 [21]	6.36 [114]
propranolol	5.68 [21]	4.74 [21]	5.55 [115]
quetiapine	4.77 [21]	4.98 [21]	5.24 [116]
quinidine	4.78 [21]	4.81 [21]	6.52 [117]
risperidone	3.99 [21]	4.14 [21]	6.82 [101]
sertindole	5.64 [118]	5.05 [118]	7.85 [101]
sotalol	n/a [119]	n/a [119]	7.07 [119]
tedisamil	4.70 [120]	n/a [121]	5.60 [101]
terfenadine	6.01 [21]	6.43 [21]	8.05 [115]
thioridazine	5.74 [21]	5.89 [21]	7.48 [101]
verapamile	4.38 [21]	7.00 [21]	6.84 [97]

## Abbreviations

VGICs	Voltage-Gated Ion Channels
VGKCs	Voltage-Gated Potassium Channels
VGNaCs	Voltage-Gated Sodium Channels
VGCaCs	Voltage-Gated Calcium Channels
hERG	Human Ether-à-go-go-Related Gene
TdP	Torsade the points
CiPA	Comprehensive In Vitro Proarrhythmia Assay
IC_50_	Inhibition potency
K_i_	Binding affinity

## References

[1] OECD Test No. 453: Combined Chronic Toxicity/Carcinogenicity Studies, OECD Guidelines for the Testing of Chemicals, Section 4. https://www.oecd-ilibrary.org/environment/test-no-453-combined-chronic-toxicity-carcinogenicity-studies_9789264071223-en.

[2] OECD T.G.G. (1960). The Organisation for Economic Co-operation and Development.

[3] Raghavan M., Fee D., Barkhaus P.E. (2019). Generation and propagation of the action potential. Neurology of Sexual and Bladder Disorders.

[4] Alberts B., Johnson A., Lewis J., Raff M., Roberts K., Walter P. (2015). Molecular Biology of the Cell.

[5] Issa Z.F., Miller J.M., Zipes D.P. (2019). Clinical Arrhythmology and Electrophysiology: A Companion to Braunwald’s Heart Disease. Clinical Arrhythmology and Electrophysiology: A Companion to Braunwald’s Heart Disease.

[6] Giegel D., Lewis A., Worland P. (2007). Diversity versus Focus in Choosing Targets and Therapeutic Areas. Comprehensive Medicinal Chemistry II..

[7] Rubaiy H.N. (2017). A Short Guide to Electrophysiology and Ion Channels. J. Pharm. Pharm. Sci..

[8] Gingrich K.J., Yang J. (2006). Molecular physiology. Foundations of Anesthesia: Basic Sciences for Clinical Practice.

[9] Kirsch G.E., Kramer J., Bruening-Wright A., Obejero-Paz C., Brown A.M. (2016). The Comprehensive In Vitro Proarrhythmia Assay (CiPA) Guide: A New Approach to Cardiac Risk Assessment.

[10] Li Z., Ridder B.J., Han X., Wu W.W., Sheng J., Tran P.N., Wu M., Randolph A., Johnstone R.H., Mirams G.R. (2019). Assessment of an In Silico Mechanistic Model for Proarrhythmia Risk Prediction Under the Ci PA Initiative. Clin. Pharmacol. Ther..

[11] Raschi E., Vasina V., Poluzzi E., de Ponti F. (2008). The hERG K+ channel: Target and antitarget strategies in drug development. Pharmacol. Res..

[12] Petkov G.V. (2009). Ion channels. Pharmacology.

[13] Cheung S., Parkinson J., Wählby-Hamrén U., Dota C.D., Kragh A.M., Bergenholm L., Vik T., Collins T., Arfvidsson C., Pollard C.E. (2018). A tutorial on model informed approaches to cardiovascular safety with focus on cardiac repolarisation. J. Pharmacokinet. Pharmacodyn..

[14] Sallam K., Li Y., Sager P.T., Houser S.R., Wu J.C. (2015). Finding the Rhythm of Sudden Cardiac Death. Circ. Res..

[15] Lee H.-M., Yu M.-S., Kazmi S.R., Oh S.Y., Rhee K.-H., Bae M.-A., Lee B.H., Shin D.-S., Oh K.-S., Ceong H. (2019). Computational determination of hERG-related cardiotoxicity of drug candidates. BMC Bioinform..

[16] Woosley R.D., Romero K., Heise C.W., Gallo T., Tate J., Woosley R.L. (2019). Summary of Torsades de Pointes (TdP) Reports Associated with Intravenous Drug Formulations Containing the Preservative Chlorobutanol. Drug Saf..

[17] (2005). Guidance for Industry: E14 Clinical Evaluation of QT/QTc Interval Prolongation and Proarrhythmic Potential for Non-Antiarrhythmic Drugs.

[18] (2015). ICH E14 Guideline: The Clinical Evaluation of QT/QTc Interval Prolongation and Proarrhythmic Potential for Non-Antiarrhythmic Drugs.

[19] Li Z., Dutta S., Sheng J., Tran P.N., Wu W., Chang K., Mdluli T., Strauss D.G., Colatsky T. (2017). Improving the In Silico Assessment of Proarrhythmia Risk by Combining hERG (Human Ether-à-go-go-Related Gene) Channel–Drug Binding Kinetics and Multichannel Pharmacology. Circ. Arrhythmia Electrophysiol..

[20] Hondeghem L., Carlsson L., Duker G. (2001). Instability and Triangulation of the Action Potential Predict Serious Proarrhythmia, but Action Potential Duration Prolongation Is Antiarrhythmic. Circulation.

[21] Mirams G.R., Cui Y., Sher A., Fink M., Cooper J., Heath B.M., McMahon N.C., Gavaghan D.J., Noble D. (2011). Simulation of multiple ion channel block provides improved early prediction of compounds’ clinical torsadogenic risk. Cardiovasc. Res..

[22] Martin R.L., McDermott J.S., Salmen H.J., Palmatier J., Cox B.F., Gintant G.A. (2004). The Utility of hERG and Repolarization Assays in Evaluating Delayed Cardiac Repolarization: Influence of Multi-Channel Block. J. Cardiovasc. Pharmacol..

[23] Blinova K., Stohlman J., Vicente J., Chan D., Johannesen L., Hortigon-Vinagre M.P., Zamora V., Smith G., Crumb W.J., Pang L. (2016). Comprehensive Translational Assessment of Human-Induced Pluripotent Stem Cell Derived Cardiomyocytes for Evaluating Drug-Induced Arrhythmias. Toxicol. Sci..

[24] Sager P.T., Gintant G., Turner J.R., Pettit S., Stockbridge N. (2014). Rechanneling the cardiac proarrhythmia safety paradigm: A meeting report from the Cardiac Safety Research Consortium. Am. Hear. J..

[25] Barker B.S., Young G.T., Soubrane C.H., Stephens G.J., Stevens E.B., Patel M.K. (2017). Chapter 2—Ion Channels. Conn’s Translational Neuroscience.

[26] Dunlop J., Bowlby M.R., Peri R., Vasilyev D., Arias R. (2008). High-throughput electrophysiology: An emerging paradigm for ion-channel screening and physiology. Nat. Rev. Drug Discov..

[27] Harmar A.J., Hills R.A., Rosser E.M., Jones M., Buneman O.P., Dunbar D.R., Greenhill S.D., Hale V.A., Sharman J.L., Bonner T.I. (2008). IUPHAR-DB: The IUPHAR database of G protein-coupled receptors and ion channels. Nucleic Acids Res..

[28] Eranjan R., Logette E., Marani M., Herzog M., Tâche V., Scantamburlo E., Buchillier V., Markram H. (2019). A Kinetic Map of the Homomeric Voltage-Gated Potassium Channel (Kv) Family. Front. Cell. Neurosci..

[29] Bennett D.L.H., Woods C.G. (2014). Painful and painless channelopathies. Lancet Neurol..

[30] Allen N.M., Weckhuysen S., Gorman K., King M.D., Lerche H. (2020). Genetic potassium channel-associated epilepsies: Clinical review of the Kv family. Eur. J. Paediatr. Neurol..

[31] Fernández-Ballester G., Fernández-Carvajal A., González-Ros J.M., Ferrer-Montiel A. (2011). Ionic Channels as Targets for Drug Design: A Review on Computational Methods. Pharmaceutics.

[32] Amin A.S., Tan H.L., Wilde A.A. (2010). Cardiac ion channels in health and disease. Hear. Rhythm..

[33] Wulff H., Castle N.A., Pardo L.A. (2009). Voltage-gated potassium channels as therapeutic targets. Nat. Rev. Drug Discov..

[34] Abbott G.W., Sesti F., Splawski I., Buck M., Lehmann M.H., Timothy K.W., Keating M.T., Goldstein S.A. (1999). MiRP1 Forms I Kr Potassium Channels with HERG and is Associated with Cardiac Arrhythmia. Cell.

[35] Isbrandt D., Friederich P., Solth A., Haverkamp W., Ebneth A., Borggrefe M., Funke H., Sauter K., Breithardt G., Pongs O. (2002). Identification and functional characterization of a novel KCNE2 (MiRP1) mutation that alters HERG channel kinetics. J. Mol. Med..

[36] Hu B., Zeng W.-P., Li X., Al-Sheikh U., Chen S.-Y., Ding J. (2019). A conserved arginine/lysine-based motif promotes ER export of KCNE1 and KCNE2 to regulate KCNQ1 channel activity. Channels.

[37] Alexander S.P.H., Fabbro D., Kelly E., Mathie A., Peters j., Veale E.L., Armstrong J.F., Faccenda E., Harding S.D., Pawson A.J. (2019). The Concise Guide to Pharmacology 2019/20: Enzymes. Br. J. Pharmacol..

[38] Huang H., Pugsley M.K., Fermini B., Curtis M.J., Koerner J., Accardi M., Authier S. (2017). Cardiac voltage-gated ion channels in safety pharmacology: Review of the landscape leading to the CiPA initiative. J. Pharmacol. Toxicol. Methods.

[39] Wang W., MacKinnon R. (2017). Cryo-EM Structure of the Open Human Ether-à-go-go-Related K + Channel hERG. Cell.

[40] Mobasheri A., Matta C., Uzieliene I., Budd E., Martín-Vasallo P., Bernotiene E. (2019). The chondrocyte channelome: A narrative review. Jt. Bone Spine.

[41] Vicente J., Zusterzeel R., Johannesen L., Mason J., Sager P., Patel V., Matta M.K., Philip S., Liu J., Garnett C. (2017). Mechanistic Model-Informed Proarrhythmic Risk Assessment of Drugs: Review of the “CiPA” Initiative and Design of a Prospective Clinical Validation Study. Clin. Pharmacol. Ther..

[42] Orvos P., Kohajda Z., Szlovák J., Gazdag P., Árpádffy-Lovas T., Tóth D., Geramipour A., Tálosi L., Jost N., Varró A. (2018). Evaluation of Possible Proarrhythmic Potency: Comparison of the Effect of Dofetilide, Cisapride, Sotalol, Terfenadine, and Verapamil on hERG and Native IKr Currents and on Cardiac Action Potential. Toxicol. Sci..

[43] Ajayi F., Sun H., Perry J. (2000). Adverse drug reactions: A review of relevant factors. J. Clin. Pharm..

[44] Li M., Ramos L.G. (2017). Drug-Induced QT Prolongation and Torsades de Pointes. Pharm. Therap..

[45] Ringer S. (1883). A further contribution regarding the influence of the different constituents of the blood on the contraction of the heart. J. Physiol..

[46] Heilbrunn L.V., Wiercinski F.J. (1947). The action of various cations on muscle protoplasm. J. Cell. Comp. Physiol..

[47] Cosconati S., Marinelli L., Lavecchia A., Novellino E. (2007). Characterizing the 1,4-Dihydropyridines Binding Interactions in the L-Type Ca^2+^ Channel: Model Construction and Docking Calculations. J. Med. Chem..

[48] Zwanzger P., Eßer D., Nothdurfter C., Baghai T.C., Möller H.-J., Padberg F., Rupprecht R. (2009). Effects of the GABA-reuptake Inhibitor Tiagabine on Panic and Anxiety in Patients with Panic Disorder. Pharmacopsychiatry.

[49] Li P., Rubaiy H.N., Chen G., Hallett T., Zaibi N., Zeng B., Saurabh R., Xu S. (2020). T-type Ca2+ channel blocker mibefradil inhibits ORAI store-operated channels. J. Mol. Cell. Cardiol..

[50] García A.G., García-De-Diego A.M., Gandia L., Borges R., García-Sancho J. (2006). Calcium Signaling and Exocytosis in Adrenal Chromaffin Cells. Physiol. Rev..

[51] Zamponi G.W., Striessnig J., Koschak A., Dolphin A.C. (2015). The Physiology, Pathology, and Pharmacology of Voltage-Gated Calcium Channels and Their Future Therapeutic Potential. Pharmacol. Rev..

[52] Hofmann F., Flockerzi V., Kahl S., Wegener J.W. (2014). L-Type CaV1.2 Calcium Channels: From In Vitro Findings to In Vivo Function. Physiol. Rev..

[53] Balasubramanian B., Imredy J.P., Kim D., Penniman J., Lagrutta A., Salata J.J. (2009). Optimization of Cav1.2 screening with an automated planar patch clamp platform. J. Pharmacol. Toxicol. Methods.

[54] Striessnig J., Pinggera A., Kaur G., Bock G., Tuluc P. (2014). L-type Ca^2+^ channels in heart and brain. Wiley Interdiscip. Rev. Membr. Transp. Signal..

[55] Théophile G. (2017). Discovery and Development of Calcium Channel Blockers. Front. Pharmacol..

[56] Reed J.K., Raftery M.A. (1976). Properties of the tetrodotoxin binding component in plasma membranes isolated from Electrophorus electricus. Biochemistry.

[57] Chahine M. (2018). Voltage-gated Sodium Channels: Structure, Function and Channelopathies.

[58] Wiffen P.J., Moore R.A., Aldington D., Cole P., Rice A.S., Lunn M.P., Hamunen K., Haanpää M., Kalso E., Derry S. (2013). Antiepileptic drugs for neuropathic pain and fibromyalgia—An overview of Cochrane reviews. Cochrane Database Syst. Rev..

[59] de Marco K.R., Clancy C.E. (2016). Cardiac Na Channels: Structure to Function. Curr. Top. Membr..

[60] Mantegazza M., Curia G., Biagini G., Ragsdale D.S., Avoli M. (2010). Voltage-gated sodium channels as therapeutic targets in epilepsy and other neurological disorders. Lancet Neurol..

[61] Bagnéris C., DeCaen P.G., Naylor C.E., Pryde D.C., Nobeli I., Clapham D.E., Wallace B.A. (2014). Prokaryotic NavMs channel as a structural and functional model for eukaryotic sodium channel antagonism. Proc. Natl. Acad. Sci. USA.

[62] Yan Z., Zhou Q., Wang L., Wu J., Zhao Y., Huang G., Peng W., Shen H., Lei J., Yan N. (2017). Structure of the Na v 1.4-β1 Complex from Electric Eel. Cell.

[63] Li Z., Jin X., Huang G., Wu K., Lei J., Pan X., Yan N. (2019). Structural basis for pore blockade of the human cardiac sodium channel Nav1.5 by tetrodotoxin and quinidine. bioRxiv.

[64] Huang W., Liu M., Yan S.F., Yan N. (2017). Structure-based assessment of disease-related mutations in human voltage-gated sodium channels. Prot. Cell.

[65] Loussouarn G., Sternberg D., Nicole S., Marionneau C., le Bouffant F., Toumaniantz G., Barc J., Malak O.A., Fressart V., Péreon Y. (2016). Physiological and Pathophysiological Insights of Nav1.4 and Nav1.5 Comparison. Front. Pharmacol..

[66] Dib-Hajj S.D., Yang Y., Black J.A., Waxman S.G. (2012). The NaV1.7 sodium channel: From molecule to man. Nat. Rev. Neurosci..

[67] Theile J.W., Cummins T.R. (2011). Recent Developments Regarding Voltage-Gated Sodium Channel Blockers for the Treatment of Inherited and Acquired Neuropathic Pain Syndromes. Front. Pharmacol..

[68] Du Y., Garden D.P., Wang L., Zhorov B.S., Dong K. (2011). Identification of New Batrachotoxin-sensing Residues in Segment IIIS6 of the Sodium Channel. J. Biol. Chem..

[69] Żelazny P., Uździcki A., Awgul S., Pawlik A. (2018). Kanały jonowe jako punkty uchwytu leków stosowanych w terapii padaczki. Farm. Współczesna.

[70] Lei M., Wu L., Terrar D.A., Huang C.L.-H. (2018). Modernized Classification of Cardiac Antiarrhythmic Drugs. Circulation.

[71] Yellen G. (1998). The moving parts of voltage-gated ion channels. Q. Rev. Biophys..

[72] Chang C., Ray A., Swaan P. (2005). In silico strategies for modeling membrane transporter function. Drug Discov. Today.

[73] Radchenko E., Rulev Y.A., Safanyaev A.Y., Palyulin V., Zefirov N. (2017). Computer-aided estimation of the hERG-mediated cardiotoxicity risk of potential drug components. Dokl. Biochem. Biophys..

[74] Recanatini M., Poluzzi E., Masetti M., Cavalli A., De Ponti F. (2005). QT Prolongation Through hERG K+ Channel Blockade: Current Knowledge and Strategies for the Early Prediction During Drug Development. Med. Res. Rev..

[75] Huang S.-M., Temple R., Throckmorton D.C., Lesko L.J. (2007). Drug Interaction Studies: Study Design, Data Analysis, and Implications for Dosing and Labeling. Clin. Pharmacol. Ther..

[76] Zaręba P., Gryzło B., Malawska K., Sałat K., Höfner G.C., Nowaczyk A., Fijałowski Ł., Rapacz A., Podkowa A., Furgała A. (2020). Novel GABA uptake inhibitors with enhanced inhibitory activity toward mGAT3/4 and their effect on pain threshold in mice. EJMCh.

[77] Böck M.C., Höfner G., Wanner K.T. (2020). N-Substituted Nipecotic Acids as (S)-SNAP-5114 Analogues with Modified Lipophilic Domains. ChemMedChem.

[78] Nowaczyk A., Fijałkowski Ł., Zaręba P., Sałat K. (2018). Selective neuronal and astrocytic inhibition of human GABA transporter isoform 1 (hGAT1) inhibitors in the mechanism of epilepsy and pain-molecular docking and pharmacodynamics studies, part I. J. Mol. Grap. Model..

[79] Bahnikova M., Matejoviâ P., Pasek M., Imurdová M., Imurda J. (2002). Ajmaline-induced block of sodium current in rat ventricular myocytes. Scr. Med..

[80] Bébarová M., Matejovic P., Pásek M., Simurdová M., Simurda J. (2005). Effect of ajmaline on action potential and ionic currents in rat ventricular myocytes. Gen. Physiol. Biophys..

[81] Kiesecker C., Zitron E., Bloehs R., Scholz E., Thomas D., Kreye V.A.W., Katus H.A., Schoels W., Karle C.A., Kiehn J. (2004). Class Ia anti-arrhythmic drug ajmaline blocks HERG potassium channels: Mode of action. Naunyn Schmiedeberg’s Arch. Pharmacol..

[82] Grima M., Schwartz J., Spach M., Velly J. (1986). Anti-anginal arylalkylamines and sodium channels: [3H]-batrachotoxinin-A 20-alpha-benzoate and [3H]-tetracaine binding. Br. J. Pharmacol..

[83] Lubic S.P., Nguyen K.P., Dave B., Giacomini J.C. (1994). Antiarrhythmic agent amiodarone possesses calcium channel blocker properties. J. Cardiovasc. Pharmacol..

[84] Leffler A., Reiprich A., Mohapatra D.P., Nau C. (2007). Use-dependent block by lidocaine but not amitriptyline is more pronounced in tetrodotoxin (TTX)-Resistant Nav1. 8 than in TTX-sensitive Na+ channels. J. Pharmacol. Exp. Ther..

[85] Zahradník I., Minarovič I., Zahradníková A. (2008). Inhibition of the cardiac L-type calcium channel current by antidepressant drugs. J. Pharmacol. Exp. Ther..

[86] Jo S.H., Youm J.B., Lee C.O., Earm Y.E., Ho W.K. (2000). Blockade of the HERG human cardiac K+ channel by the antidepressant drug amitriptyline. Br. J. Pharmacol..

[87] Fossa A.A., de Pasquale M.J., Raunig D.L., Avery M.J., Leishman D.J. (2002). The relationship of clinical QT prolongation to outcome in the conscious dog using a beat-to-beat QT-RR interval assessment. J. Pharmacol. Exp. Ther..

[88] McNeal E.T., Lewandowski G.A., Daly J.W., Creveling C. (1985). [3H] Batrachotoxinin A 20. alpha.-benzoate binding to voltage-sensitive sodium channels: A rapid and quanitative assay for local anesthetic activity in a variety of drugs. J. Med. Chem..

[89] Testa R., Abbiati G., Ceserani R., Restelli G., Vanasia A., Barone D., Gobbi M., Mennini T. (1989). Profile of in vitro binding affinities of neuroleptics at different rat brain receptors: Cluster analysis comparison with pharmacological and clinical profiles. Pharm. Res..

[90] Sato T., Wu B., Kiyosue T., Arita M. (1994). Effects of cibenzoline, a new class Ia antiarrhythmic drug, on various membrane ionic currents and action potentials of guinea-pig ventricular cells. Naunyn Schmiedeberg’s Arch. Pharmacol.

[91] Matsuoka S., Nawada T., Hisatome I., Miyamoto J., Hasegawa J., Kotake H., Mashiba H. (1991). Comparison of Ca2+ channel inhibitory effects of cibenzoline with verapamil on guinea-pig heart. Gen. Pharmacol. Vasc. Syst..

[92] Männikkö R., Overend G., Perrey C., Gavaghan C., Valentin J.P., Morten J., Armstrong M., Pollard C. (2010). Pharmacological and electrophysiological characterization of nine, single nucleotide polymorphisms of the hERG-encoded potassium channel. Br. J. Pharmacol..

[93] Mohammad S., Zhou Z., Gong Q., January C.T. (1997). Blockage of the HERG human cardiac K+ channel by the gastrointestinal prokinetic agent cisapride. Am. J. Physiol. Content.

[94] Ekins S., Crumb W.J., Sarazan R.D., Wikel J.H., Wrighton S.A. (2002). “Three dimensional quantitative structure activity relationship for the inhibition of the hERG (human ether-a-gogo related gene) potassium channel,”. J. Pharmacol. Exp. Thera..

[95] Pauwels P.J., Laduron P.M. (1986). TPP+ accumulation in rat brain synaptosomes as a probe for Na+ channels. Eur. J. Pharmacol..

[96] Campiani G., Fiorini I., de Filippis M.P., Ciani S.M., Garofalo A., Nacci V., Giorgi G., Sega A., Botta M., Chiarini A. (1996). Cardiovascular characterization of pyrrolo [2, 1-d][1, 5] benzothiazepine derivatives binding selectively to the peripheral-type benzodiazepine receptor (PBR): From dual PBR affinity and calcium antagonist activity to novel and selective calcium entry blockers. J. Med. Chem..

[97] Zhang S., Zhou Z., Gong Q., Makielski J.C., January C.T. (1999). Mechanism of block and identification of the verapamil binding domain to HERG potassium channels. Circ. Res..

[98] Johnson R.E., Silver P.J., Becker R., Birsner N.C., Bohnet E.A., Briggs G.M., Busacca C.A., Canniff P., Carabateas P.M., D’Ambra T. (1995). 4, 5-Dihydro-3-(methanesulfonamidophenyl)-1-phenyl-1H-2, 4-benzodiazepines: A novel class III antiarrhythmic agents. J. Med. Chem..

[99] Chadwick C.C., Ezrin A.M., O’Connor B., Volberg W.A., Smith D.I., Wedge K.J., Hill R.J., Briggs G.M., Pagani E.D., Silver P.J. (1993). Identification of a specific radioligand for the cardiac rapidly activating delayed rectifier K+ channel. Circ. Res..

[100] de Bruin M., Pettersson M., Meyboom R., Hoes A., Leufkens H. (2005). Anti-HERG activity and the risk of drug-induced arrhythmias and sudden death. Eur. Heart J..

[101] Redfern W., Carlsson L., Davis A., Lynch W., MacKenzie I., Palethorpe S., Siegl P., Strang I., Sullivan A., Wallis R. (2003). Relationships between preclinical cardiac electrophysiology, clinical QT interval prolongation and torsade de pointes for a broad range of drugs: Evidence for a provisional safety margin in drug development. Cardiovasc. Res..

[102] Schoemaker H., Claustre Y., Fage D., Rouquier L., Chergui K., Curet O., Oblin A., Gonon F., Carter C., Benavides J. (1997). Neurochemical characteristics of amisulpride, an atypical dopamine D2/D3 receptor antagonist with both presynaptic and limbic selectivity. J. Pharmacol. Exp. Therap..

[103] Kirsch G.E., Trepakova E.S., Brimecombe J.C., Sidach S.S., Erickson H.D., Kochan M.C., Shyjka L.M., Lacerda A.E., Brown A.M. (2004). Variability in the measurement of hERG potassium channel inhibition: Effects of temperature and stimulus pattern. J. Pharmacol. Toxicol. Methods.

[104] de Luca A., Talon S., De Bellis M., Desaphy J.-F., Franchini C., Lentini G., Catalano A., Corbo F., Tortorella V., Conte-Camerino D. (2003). Inhibition of skeletal muscle sodium currents by mexiletine analogues: Specific hydrophobic interactions rather than lipophilia per se account for drug therapeutic profile. Naunyn Schmiedeberg’s Arch. Pharmacol..

[105] Hu X., Qian J. (2001). DDPH inhibited L-type calcium current and sodium current in single ventricular myocyte of guinea pig. Acta Pharmacol. Sin..

[106] Roche O., Trube G., Zuegge J., Pflimlin P., Alanine A., Schneider G. (2002). A virtual screening method for prediction of the HERG potassium channel liability of compound libraries. ChemBioChem.

[107] Strege P.R., Bernard C.E., Ou Y., Gibbons S.J., Farrugia G. (2005). Effect of mibefradil on sodium and calcium currents. Am. J. Physiol. Liver Physiol..

[108] Huber I., Wappl E., Herzog A., Mitterdorfer J., Glossmann H., Langer T., Striessnig J. (2000). Conserved Ca2+-antagonist-binding properties and putative folding structure of a recombinant high-affinity dihydropyridine-binding domain. Biochem. J..

[109] di Stilo A., Visentin S., Cena C., Gasco A.M., Ermondi G., Gasco A. (1998). New 1, 4-dihydropyridines conjugated to furoxanyl moieties, endowed with both nitric oxide-like and calcium channel antagonist vasodilator activities. J. Med. Chem..

[110] Zhabyeyev P., Missan S., Jones S.E., McDonald T.F. (2000). Low-affinity block of cardiac K+ currents by nifedipine. Eur. J. Pharmacol..

[111] Ehlert F.J., Roeske W.R., Itoga E., Yamamura H.I. (1982). The binding of [3H] nitrendipine to receptors for calcium channel antagonists in the heart, cerebral cortex, and ileum of rats. Life Sci..

[112] Pauwels P.J., Leysen J.E., Laduron P.M. (1986). [3H] Batrachotoxinin A 20-α-benzoate binding to sodium channels in rat brain: Characterization and pharmacological significance. Eur. J. Pharmacol..

[113] Reynolds I., Snowman A.M., Snyder S.H. (1986). (-)-[3H] desmethoxyverapamil labels multiple calcium channel modulator receptors in brain and skeletal muscle membranes: Differentiation by temperature and dihydropyridines. J. Pharmacol. Exp. Ther..

[114] Paul A.A., Witchel H.J., Hancox J.C. (2002). Inhibition of the current of heterologously expressed HERG potassium channels by flecainide and comparison with quinidine, propafenone and lignocaine. Br. J. Pharmacol..

[115] Hanson L.A., Bass A.S., Gintant G., Mittelstadt S., Rampe D., Thomas K. (2006). ILSI-HESI cardiovascular safety subcommittee initiative: Evaluation of three non-clinical models of QT prolongation. J. Pharmacol. Toxicol. Methods.

[116] Kongsamut S., Kang J., Chen X.-L., Roehr J., Rampe D. (2002). A comparison of the receptor binding and HERG channel affinities for a series of antipsychotic drugs. Eur. J. Pharmacol..

[117] Po S., Wang D., Yang I.C.-H., Johnson J., Nie L., Bennett P. (1999). Modulation of HERG potassium channels by extracellular magnesium and quinidine. J. Cardiovasc. Pharmacol..

[118] Thomsen M.B., Volders P.G., Stengl M., Späatjens R.L., Beekman J.D., Bischoff U., Kall M.A., Frederiksen K., Matz J., Vos M.A. (2003). Electrophysiological safety of sertindole in dogs with normal and remodeled hearts. J. Pharmacol. Exp. Ther..

[119] Kramer J., Obejero-Paz C.A., Myatt G., Kuryshev Y.A., Bruening-Wright A., Verducci J.S., Brown A.M. (2013). MICE models: Superior to the HERG model in predicting Torsade de Pointes. Sci. Rep..

[120] Faivre J.F., Gout B., Bril A. (1995). Tedisamil. Card. Drug Rev..

[121] Jost N., Virág L., Hála O., Varro A., Thormahlen D., Papp J.G. (2004). Effect of the antifibrillatory compound tedisamil (KC-8857) on transmembrane currents in mammalian ventricular myocytes. Curr. Med. Chem..

